# Drought legacy in mature spruce alleviates physiological stress during recurrent drought

**DOI:** 10.1111/plb.70039

**Published:** 2025-05-16

**Authors:** K. Hikino, B. D. Hesse, T. Gebhardt, B. D. Hafner, C. Buchhart, M. Baumgarten, K.‐H. Häberle, T. E. E. Grams

**Affiliations:** ^1^ School of Life Sciences, Land Surface‐Atmosphere Interactions, Ecophysiology of Plants Technical University of Munich Freising Germany; ^2^ Department of Forest Ecology and Management Swedish University of Agricultural Sciences (SLU) Umeå Sweden; ^3^ Department of Integrative Biology and Biodiversity Research, Institute of Botany University of Natural Resources and Life Sciences Vienna Austria; ^4^ School of Life Sciences, Forest and Agroforest Systems Technical University of Munich Freising Germany; ^5^ School of Life Sciences, Soil Biophysics & Environmental Systems Technical University of Munich Freising Germany; ^6^ School of Life Sciences, Chair of Restoration Ecology Technical University of Munich Freising Germany; ^7^ Present address: TU Dresden, Institute of Soil Science and Site Ecology Pienner Strasse 19, Tharandt 01737 Germany

**Keywords:** Acclimation, climate change, drought recovery, *Fagus sylvatica*, leaf gas exchange, *Picea abies*, water potential, xylem sap flow density

## Abstract

- Forest ecosystems are facing severe and prolonged droughts with delayed recovery, known as “drought legacy”. This study presents positive legacy effects following a long‐term, experimental drought and subsequent recovery in a mature mixed Norway spruce and European beech forest.
- Approximately 50 mature trees were exposed to five consecutive years of summer drought by completely excluding growing season precipitation from May 2014 to June 2019. Experimental drought recovery started in July 2019, after which the trees received natural precipitation. Taking advantage of the natural summer drought of 2022, following the unique long‐term experimental drought, we investigated how drought legacy affects tree physiological responses to recurrent drought.
- The long‐term experimental drought resulted in a 60% reduction in spruce leaf area, which was still reduced by 30% 4 years after the drought release. This slow recovery and associated reduced water use resulted in higher soil water availability under spruce during the 2022 drought, leading to significantly reduced physiological drought stress: about two times higher predawn leaf water potential, leaf gas exchange and sap flow density in legacy spruce compared to previous controls. Furthermore, neighbouring beech, displaying no leaf area reduction during the experimental drought, also had higher predawn leaf water potential and leaf gas exchange during the 2022 drought compared to previous controls, likely benefitting from the reduced water use of spruce.
- The slow recovery of spruce leaf area as a pronounced drought legacy effect proved advantageous for trees in alleviating physiological stress and overcoming future drought events.

## Graphical abstract

The slow recovery of leaf area in mature spruce following a long‐term drought alleviated physiological stress during recurrent drought.

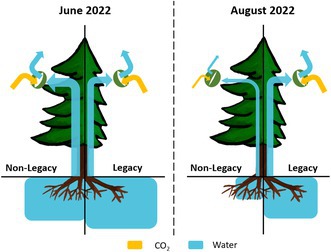

## INTRODUCTION

Forest ecosystems have a discernible impact on global climate by exchanging carbon and water with the surrounding atmosphere (Brüggemann *et al*. 2011; Ellison *et al*. 2017). Forests have been suffering from severe and frequent drought events, globally causing large areas of tree and stand mortality (Allen *et al*. 2010; Hartmann *et al*. 2018; Schuldt *et al*. 2020; Hammond *et al*. 2022). Frequency and duration of drought events are predicted to increase (IPCC 2021). Drought decreases tree water use, carbon uptake, and growth (Ciais *et al*. 2005; Peñuelas *et al*. 2011; Hartmann *et al*. 2018). However, trees are able to acclimate to changing climate conditions, leading to non‐linear morphological and physiological responses, especially under a long‐term drought (Leuzinger *et al*. 2011; Beier *et al*. 2012; Barbeta *et al*. 2013; Feichtinger *et al*. 2014; Liu *et al*. 2015).

Drought events are necessarily associated with drought recovery. Compared to drought effects, however, drought recovery processes have been much less investigated especially in mature trees (Ruehr *et al*. 2019; Vilonen *et al*. 2022). The speed of this recovery varies among different tree species and functions. For example, while leaf physiology, such as stomatal conductance, may recover within hours or days (Ruehr *et al*. 2019; Hesse *et al*. 2023), recovery of leaf morphology, such as total leaf area, can take much longer, i.e. years (Zweifel *et al*. 2020; Arend *et al*. 2022; Losso *et al*. 2022), especially in evergreen species (Song *et al*. 2022). This slow recovery of total leaf area may restrict whole tree carbon uptake, water use, and growth, even after full recovery of tree physiology. Indeed, there have been reports of delayed recovery of tree growth, known as “drought legacy” (Anderegg *et al*. 2015; Peltier *et al*. 2016; Kannenberg *et al*. 2020; Li *et al*. 2023; Miller *et al*. 2023). Repeated drought events in the past can amplify canopy dieback, mortality or reduce tree growth during another acute drought (Lloret *et al*. 2004; Mueller *et al*. 2005; Matusick *et al*. 2018; Oberleitner *et al*. 2022). However, slow or lack of recovery might also be advantageous during new drought events and can be seen as “acclimation” (Walter *et al*. 2013; Gessler *et al*. 2020). Recent studies on saplings (Tombesi *et al*. 2018; Nóia Júnior *et al*. 2020; Pritzkow *et al*. 2021; Santos *et al*. 2022) and grass species (Backhaus *et al*. 2014; Nosalewicz *et al*. 2018) show that pre‐exposure to drought increases plant resistance to the next drought, e.g. due to morphological responses such as reduction in leaf area and increased root biomass. Leaf area reduction has been shown to improve leaf physiology during drought (Gao *et al*. 2017; Ambrose *et al*. 2018; Bert *et al*. 2021; Lemaire *et al*. 2021), as water availability per leaf area increases. Since duration and frequency of drought events are predicted to further increase, trees most likely experience multiple drought events in their lifetime. Therefore, the ability to acclimate to repeated soil water‐limiting periods is essential for tree survival. It is, however, still widely unknown, how tree responses during the first drought affect tree performances during the future drought events in mature trees (Gessler *et al*. 2020; Müller & Bahn 2022).

To fill this knowledge gap, this study was conducted as part of the Kranzberg Forest roof (KROOF) project. The KROOF project was initiated in southern Germany to expose mature Norway spruce (*Picea abies* (L.) Karst) and European beech (*Fagus sylvatica* L.) trees to repeated drought by excluding precipitation throughfall during five growing seasons from May 2014 to June 2019. To initiate the recovery processes, throughfall exclusion was omitted in July 2019 and the trees received natural precipitation thereafter. During the drought period, spruce trees strongly reduced whole‐tree water use, initially through stomatal regulation and, from the third year on, by a reduction in the total leaf area (Gebhardt *et al*. 2023; Hesse *et al*. 2024). After the drought release, physiological parameters, such as phloem transport, stomatal conductance, and xylem sap flow density, fully recovered to the control level within 1 week to 1 year (Hikino *et al*. 2022; Hesse *et al*. 2023), while the total leaf area of spruce remained significantly smaller in the first years after the drought release (Gebhardt *et al*. 2023).

The fourth recovery year (2022) was an unprecedented dry year in Central Europe (Toreti *et al*. 2022; Tripathy & Mishra 2023; van der Woude *et al*. 2023). Taking advantage of this natural drought, we investigated how the legacy from the experimental long‐term drought (from May 2014 to June 2019) affected tree physiological responses during the natural summer drought in 2022. We thereby compared trees that had experienced 5 years of drought (hereafter: legacy trees) with trees on previous control plots without the drought treatment (hereafter: non‐legacy trees). After the lack of recovery in spruce leaf area within 3 years after drought release (Gebhardt *et al*. 2023), we tested the assumption that in the fourth recovery year, total leaf area was still lower in legacy trees than in the non‐legacy trees. Based on this, we hypothesized that:

1. During the natural summer drought of 2022, reduced leaf area and therefore reduced water use of legacy spruce resulted in a higher amount of relative extractable water (REW) in the legacy plots than in the non‐legacy plots.
2. Higher REW in the legacy plots led to less intense physiological stress during the summer drought of 2022 for both spruce and beech compared to trees grown on the non‐legacy plots.

## MATERIAL AND METHODS

### Experimental site

**Fig. 1:**
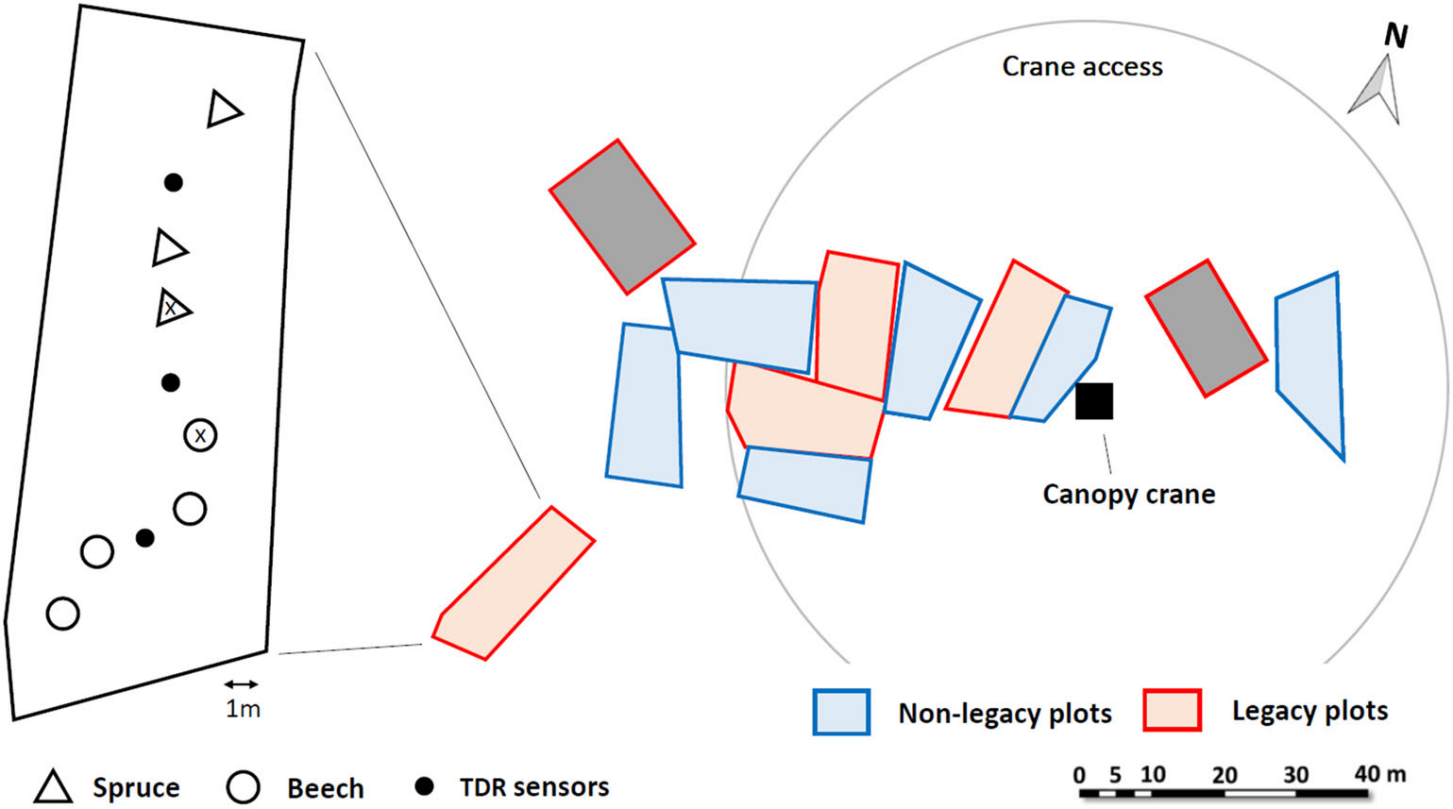
Map of the study site with non‐legacy plots (previous control plots, blue) and legacy plots (previous drought plots, red). Each plot comprises 3–6 spruce trees on one end, and 3–6 beech trees on the other end. Time Domain Reflectometry (TDR) sensors for soil water content (SWC) measurements were installed at three positions per plot (under spruce trees, between spruce and beech trees, and under beech trees). Two grey‐shaded legacy plots were excluded from the present study due to mortality through bark beetles during the drought treatment 2015: Therefore, 6 non‐legacy and 4 legacy plots were used for the study. The canopy crane provided access to tree crowns in 4 non‐legacy and 3 legacy plots for measurement of leaf physiology and morphology. Trees marked with an “x” indicate those for which average of the two TDR sensors (TDR under the respective species and TDR between beech and spruce) was used. For all other trees, REW under the corresponding species was applied.

This study was conducted at the Kranzberg Forest experimental site (close to Munich, southern Germany) in a mixed forest with ca. 90‐year‐old European beech (*F. sylvatica*) and ca. 70‐year‐old Norway spruce trees (*P. abies*) (Grams *et al*. 2021). This experimental site had a long‐term average precipitation of 750–800 mm year^−1^ and a mean air temperature of 7.8°C. The Kranzberg Forest roof (KROOF) project was initiated to expose both tree species to 5 years of repeated drought (Grams *et al*. 2021). In brief, the KROOF experimental site consists of 12 plots, each comprising of 3–6 spruce trees at one end, and 3–6 beech trees at the other end (Fig. 1). All the plots were trenched to a soil depth of about 1 m, and thick plastic tarps were installed to avoid root growth outside the plots and lateral water flow across the plots (Grams *et al*. 2021). Six plots have roofs ca. 5 m above the forest floor that close automatically during a rain event to exclude precipitation throughfall. During rain events, these roofs were closed for the entire growing seasons from May 2014 to June 2019 and, hence, 459 ± 21 mm (69 ± 7% of annual precipitation) was excluded each year. The other six plots received natural precipitation as controls. In July of the sixth year (2019), the drought treatment was omitted to investigate recovery processes (Grams *et al*. 2021; Hikino *et al*. 2022; Hikino, Danzberger, Riedel, Rehschuh, *et al*. 2022; Hesse *et al*. 2023). Initially, about 90 mm of water was supplied over 40 h to increase the soil water content of the drought plots to the control level (ca. 20%–30% of volumetric soil water content). After which, the roofs were removed and the previously drought‐stressed plots (legacy plots) received natural precipitation.

In 2015 bark beetle attack caused mortality of spruce trees on two legacy plots that were then completely excluded from the study (Grams *et al*. 2021), leaving six previous control (non‐legacy) and four legacy plots (Fig. 1). A canopy crane located in the middle of the site enabled sampling and measurements in the tree crowns of seven plots (4 non‐legacy and 3 legacy plots). Two trees of each species were chosen in each plot before the start of the drought treatment as main measurement trees, and these trees were further investigated in this study: (see Table 1 for number of replicates for each measurement parameter). Air temperature (°C), vapour pressure deficit (VPD in kPa), and precipitation (mm) were recorded by a weather station on site above the canopy.

**Table 1:** Number of replicates for each measurement parameters.

	Predawn leaf water potential	Leaf gas exchange	Sap flow density	Shoot/needle length	Total leaf area
Spruce					
Non‐legacy trees	8 (4)	8 (4)	12 (6)	8 (4)	3 (3)
Legacy trees	6 (3)	6 (3)	8 (4)	6 (3)	6 (3)
Beech					
Non‐legacy trees	8 (4)	8 (4)	n.a.	n.a.	n.a.
Legacy trees	6 (3)	6 (3)	n.a.	n.a.	n.a.

Numbers in brackets indicate number of plots.

n.a., not assessed.

### Measurements of soil water content and REW

Soil water content (SWC in vol.‐%) was measured weekly at four depths (0–7 cm, 10–30 cm, 30–50 cm, and 50–70 cm) with Time Domain Reflectometry (TDR100; Campbell Scientific, Logan, CT, USA). The sensors were installed at three positions per plot (within spruce trees, in the middle of the plots between spruce and beech trees (Mix), and within beech trees; Fig. 1 and Grams *et al*. 2021). Vol.‐% of plant available water (PAW) was then calculated for each soil layer by subtracting the permanent wilting point (threshold soil water content below which no water was available to plants; see Table S1) from SWC (Hesse *et al*. 2023). Then, the REW (%) for each soil layer was calculated using the field capacity (see Table S1):

$$\mathrm{REW}=\frac{\mathrm{PAW}}{\text{field capacity}}\times 100$$

REW for each tree was further calculated according to the tree position. For trees adjacent to the TDR sensors positioned between spruce and beech (marked with an “x” in Fig. 1 as examples), the average of REW Mix (Fig. 3b) and REW under the respective species (Fig. 3a or 3c) was used. For all other trees, REW under the corresponding species (Fig. 3a or 3c) was applied.

### Measurements of leaf gas exchange and leaf water potential

Light‐saturated stomatal conductance to water vapour (g_s_) and CO_2_ assimilation rates at a CO_2_ concentration of 400 ppm (A_sat_) were measured between 08:00 h and 14:00 h (CET) on fully sun‐exposed leaves using a LI‐6800 gas exchange system (Li‐Cor, Lincoln, NE, USA). The measurements were conducted over 2–3 sunny days in June (13–15), July (18–19), and August (16–17) 2022. Three leaves for each beech and two twigs (1‐year‐old needles) for each spruce were randomly chosen, and the average of the measurements were used for each tree. During measurements, the photosynthetic photon flux density was set to 1500 μmol m^−2^ s^−1^, relative humidity to 60%–65%, and leaf temperature was kept at 25°C. To calculate area‐based photosynthetic parameters of spruce, the needles were harvested and scanned at the end of the growing season (Epson Perfection 4990 Photo; Epson Deutschland, Meerbusch, Germany). The projected needle surface area was then multiplied by the factor 3.2 to determine the total needle surface area (Perterer & Körner 1990; Niinemets & Kull 1995). Intrinsic water use efficiency (WUE_i_) was further calculated by dividing A_sat_ by g_s_.

Predawn leaf water potential (Ψ_PD_) was measured on fully sun‐exposed twigs using a Scholander pressure bomb (model 1505D; PMS Instrument Co., Albany, OR, USA) before sunrise (03:00–05:00 h CET) on 14 June and 18 July 2022. Measurements of Ψ_PD_ could not be conducted in August due to a sudden and unexpected rain event during the night after the leaf gas exchange measurements, followed by a longer rain period.

### Measurements of xylem sap flow of spruce

Xylem sap flow density of trees was measured only in spruce. Two Granier‐type heat dissipation sensors (Granier 1987; north and south exposure) were installed in the outer xylem sapwood (0–2 cm depth) at breast height and data was logged at 10‐min intervals. Using mean values from both sensors, the mean sap flow density per day and tree was calculated (u_daily_ in L dm^−2^ day^−1^). The xylem sap flow density was analysed during 7 sunny days without rainfall around the measurement campaign of leaf water potential and leaf gas exchange in each month: 14–21 June, 14–21 July and 11–18 August 2022. The mean VPD during daytime (08:00–20:00 h) on these measurement days was 1.8–2.5 kPa (Table S2), and mean daytime photosynthetic photon flux density was 700–1000 μmol m^−2^ s^−1^.

Whole tree water use of spruce was then calculated using measured xylem sap flow density profiles (Gebhardt *et al*. 2023). The conducting sapwood depth of the measured trees was around 8 cm and did not change during the 5 years of the drought treatment and subsequent recovery (Gebhardt *et al*. 2023). The sap flow density (u_daily_) was weighted for each 1 cm of sapwood depth (i.e. 0–1 cm, 1–2, …, 7–8 cm) according to the xylem sap flow profile, multiplied by the respective sapwood area annulus, and summed to calculate whole tree daily water use (L tree^−1^ day^−1^).

### Measurements of leaf and shoot length of spruce

Needle length (mm) and shoot length (cm) of spruce trees were recorded in 4–6 randomly chosen branches per tree in sun crowns. This measurement was conducted every autumn continuously after the drought release in 2019 until the study year 2022.

### Estimation of total leaf area of spruce

The total leaf area of spruce (m^2^ tree^−1^) from 2019 to 2022 was estimated from three non‐legacy and six legacy trees, as described in detail in Gebhardt *et al*. (2023). Briefly, for each tree, the total number of needles of each needle age (*N*_n_) was calculated using field data collected on site, separately for sun and shade crowns:

$$N_{n}=N_{s}\times L_{b}\times L_{s}\times D$$

where *N*_s_ is the number of shoots of each needle age (in cm^−1^ needled branch length), *L*_b_ is total length of the needled branches (in cm), *L*_s_ is average shoot length (in cm), and *D* is average needle density (in *n* cm^−1^ shoot). *N*_s_ was counted on each tree twice after the growing season of 2020 and 2023 (see Figs. S1 and S2), on one representative branch in the middle of the sun crown (at ca. 5 m from the top) and one representative branch in the middle of the shade crown (Gebhardt *et al*. 2023). The counted number was then divided by the length of the respective branch. For *N*_s_ in 2019 and 2020, the counting data in 2020 were used. For 2022, the counting data in 2023 were used. The average of the two sets of counting data were used for *N*_s_ in 2021. Then, the total leaf area of each needle age (*A*_n_ in m^2^) was calculated using needle length (*L*_n_ in mm) following (Riederer *et al*. 1988).

$$A_{n}=\frac{N_{n}\times \left(3.279\times L_{n}-16.31\right)}{1,000,000}\;\left(\text{for current year needles}\right)$$

$$A_{n}=\frac{N_{n}\times \left(4.440\times L_{n}-24.78\right)}{1,000,000}\;\left(\text{for older needles}\right)$$

Finally, the leaf area of each needle age was summed to determine total leaf area of each tree and year.

### Statistical analysis

All data were analysed using R (v. 4.2.1) in R studio (v. 1.3.1093). Differences between non‐legacy and legacy trees were tested with a linear mixed model using the measurement campaign (months) and treatment (non‐legacy and legacy) as fixed and tree number and plot as random effects (package: nlme, v. 3.1–162). The relationships between Ψ_PD_ and REW was tested with linear regression (“lm” function, package “stats”, v. 4.2.1) with Ψ_PD_ as the dependent and REW as the independent variable. Similarly, linear regression was applied with u_daily_ as the dependent variable, and REW and VPD as independent variables. Normality of the residuals (Shapiro test) and homogeneity of variances (Levene test) was tested for every model. If any fixed factor was significant, post‐hoc test with Tukey correction (package: emmeans, v. 2.30–0) was performed. Errors in the text and graphics are presented as standard errors (SE).

## RESULTS

### Drought summer of 2022

The mean temperature between June–August 2022 was 19.7 ± 2.9°C, and the mean VPD was 0.79 ± 0.56 kPa, which were both similar to the first two recovery years (2019–2020), but 2°C higher than the long‐term mean (1999–2018; Table 2). The summed precipitation between November 2021 and August 2022 amounted to 470 mm, which was 140–280 mm lower than those of previous years (2019–2021) and the long‐term mean, marking 2022 as a drought year. A closer examination of the seasonal distribution of precipitation reveals that winter (Nov. 2021–Feb. 2022) and spring (Mar.–May 2022) precipitation were comparable to previous years, whereas summer (Jun.–Aug. 2022) precipitation was notably lower (Table S3).

**Table 2:** Mean temperature and vapour pressure deficit (VPD) from June to August, and sum of precipitation from the previous winter (November) to August, measured on the experimental site above the canopy.

	temperature (June–Aug) [°C]	VPD (June–Aug) [kPa]	precipitation (Nov–Aug) [mm]
1999–2018	17.4 ± 3.7	‐	706
2019	20.3 ± 3.4	0.85 ± 0.51	609
2020	18.1 ± 3.4	0.57 ± 0.43	643
2021	17.8 ± 3.1	0.40 ± 0.41	747
2022	19.7 ± 2.9	0.79 ± 0.56	470

Long‐term mean during 1999–2018 is taken from the nearby forest climate station (Freising, Waldklimastation, Bayerische Landesanstalt für Wald und Forstwirtschaft). Values are mean ± SD.

### Recovery of shoot length, needle length and total leaf area in spruce

**Fig. 2:**
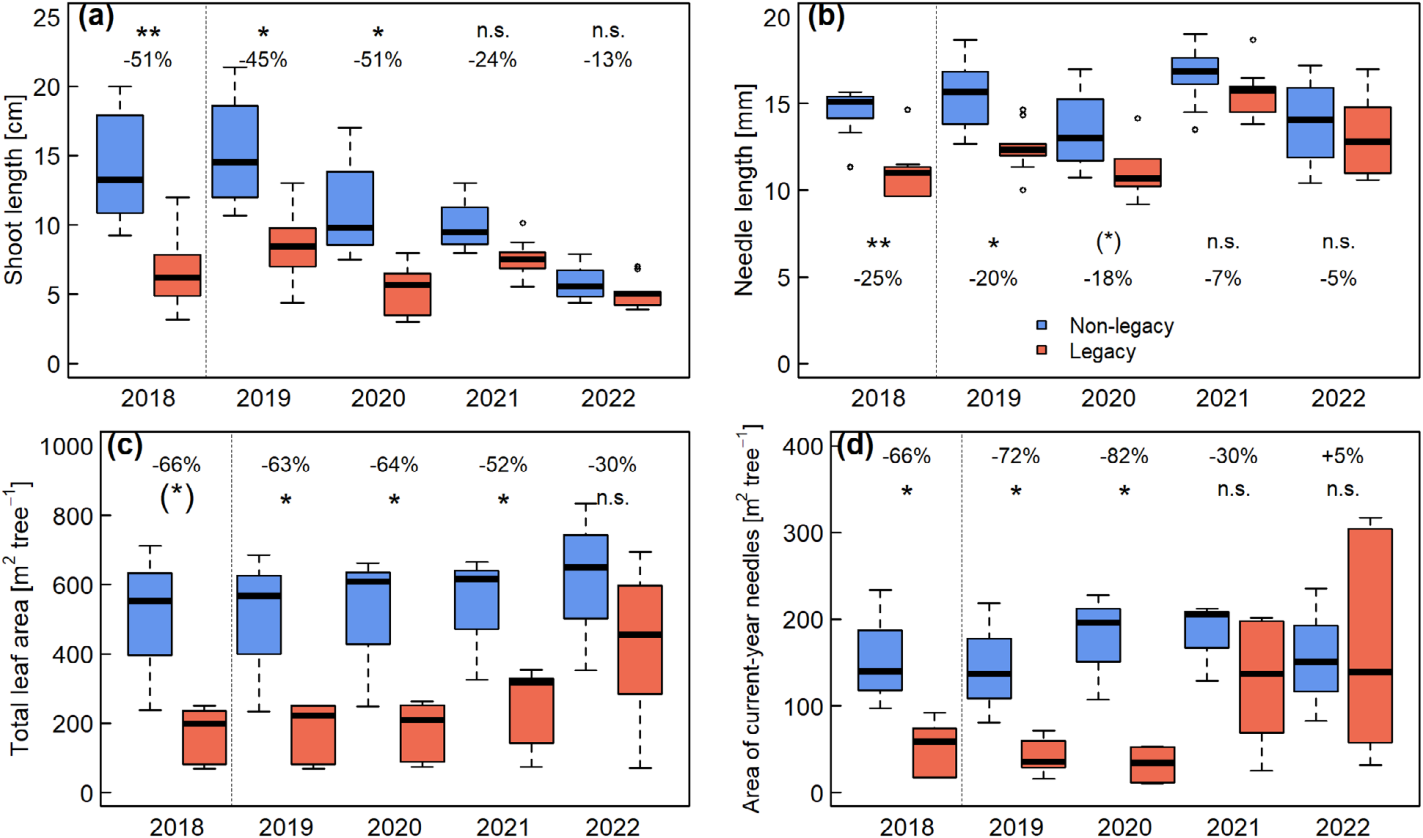
(a) Shoot length, (b) needle length, (c) total leaf area, and (d) area of current‐year needles of non‐legacy (blue) and legacy (red) spruce trees. 2018 was the last year of the drought treatment. 2022 was the study year with the natural summer drought. Asterisks indicate significant differences between non‐legacy and legacy plots according to Tukey‐HSD: **P < 0.01; *P < 0.05; (*), *P* < 0.1; n.s., not significant. Values (%) indicate differences of mean of legacy trees compared to mean of non‐legacy trees. Thick lines in boxplots represent the median.

In the first 2 years of drought recovery (2019–2020), shoot length of spruce was still significantly shorter in legacy spruce compared to the non‐legacy spruce (Fig. 2a). The legacy spruce produced shoot lengths of 8 ± 1 and 5 ± 1 cm in 2019 and 2020, which were 50% shorter (*P* < 0.05) than in those of non‐legacy spruce, at 16 ± 1 and 11 ± 1 cm, respectively. After the third recovery year, the difference between legacy and non‐legacy spruce became smaller and was not significant, mainly due to the decrease in shoot length of non‐legacy trees, especially in the drought year 2022.

In 2019 and 2020, needle length of legacy spruce was still 20% shorter (*P* < 0.05 and *P* < 0.1) than in non‐legacy spruce (Fig. 2b). From the third recovery year onwards (i.e. 2021), the needle length of legacy spruce increased to the level of non‐legacy spruce.

The total leaf area of legacy spruce was significantly smaller, by >60% in 2019 and 2020, compared to the non‐legacy spruce. In 2021, the difference between the treatments were slightly less, at 52%. In the fourth recovery year, 2022, the leaf area reduction of legacy spruce was not significant, but still 30% smaller than that of non‐legacy spruce (Fig. 2c).

The area of current‐year needles was significantly smaller in legacy spruce than in non‐legacy spruce in the last experimental drought year (2018) until the second recovery year (2020; Fig. 2d). However, from the third recovery year (2021) onwards, the area of current‐year needles of the legacy spruce recovered to the level of the non‐legacy spruce, which led to no significant difference in the total leaf area in 2022 (Fig. 2c).

### REW and predawn leaf water potential (Ψ_PD_ )

**Fig. 3:**
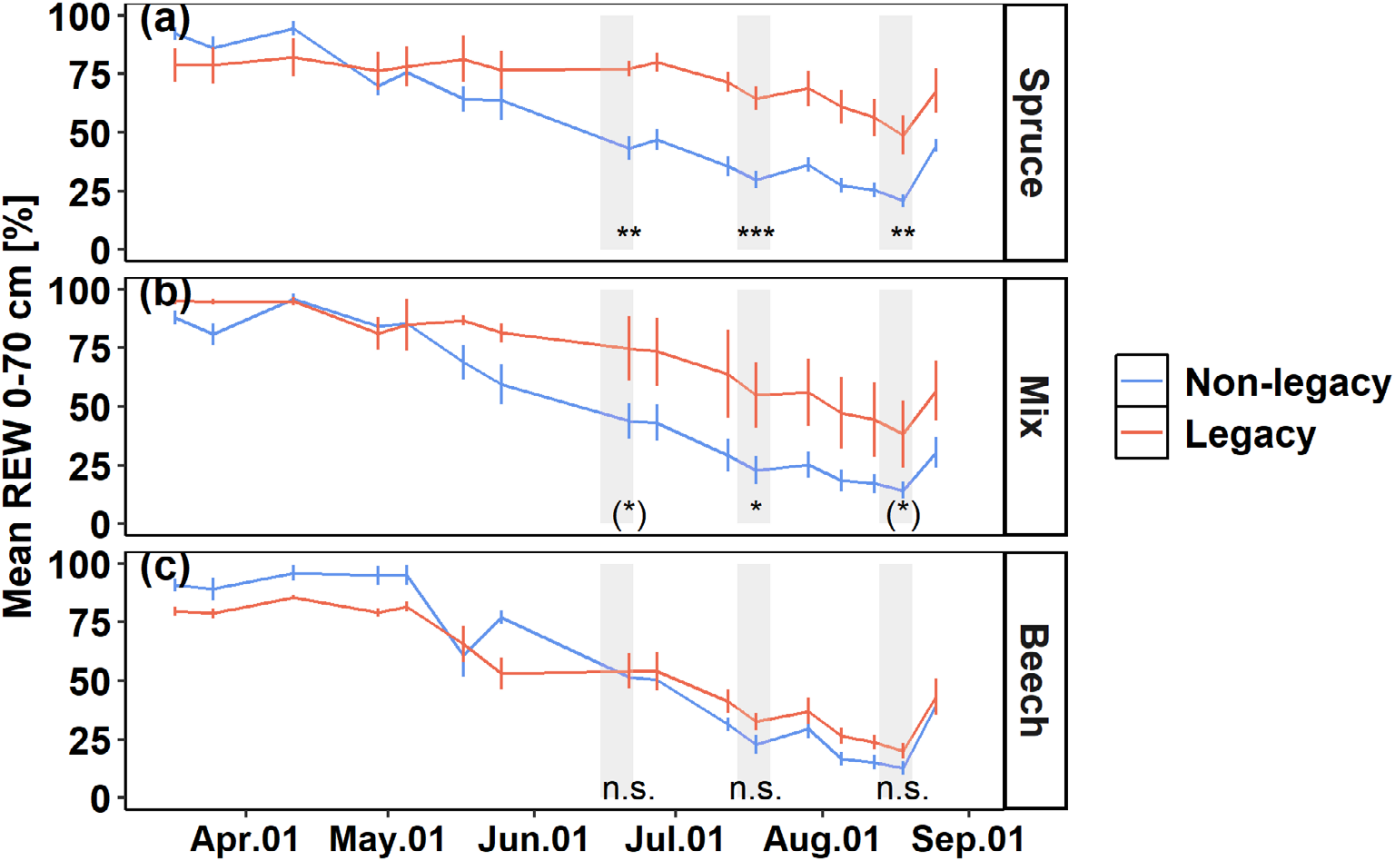
Mean relative extractable water (REW) at 0–70 cm soil depths under spruce (a), between spruce and beech (b, Mix), and under beech (c) in non‐legacy (blue) and legacy (red) plots during the measurement period 2022. Grey shaded areas are measurement days of predawn leaf water potential, leaf gas exchange, and xylem sap flow density in June, July, and August, respectively. Asterisks indicate significant differences between non‐legacy and legacy plots on measurement days according to Tukey‐HSD: *P < 0.05; (*), *P* < 0.1; n.s., not significant.

During spring, in April, REW in both non‐legacy and legacy plots was >75% in all three positions (Fig. 3), which decreased over the growing season. In the soil under spruce trees, REW was 43 ± 5% in the non‐legacy plots on 21 June (during the first measurement days of tree physiological parameters), which was only 55% of the legacy plots, at 77 ± 3% (*P* < 0.01; Fig. 3a). REW in both treatments decreased over the growing season, to 30 ± 4% and 65 ± 5% on 18 July, to 21 ± 3%, and 49 ± 8% on 18 August, respectively, where REW in the non‐legacy plots remained significantly lower than that in the legacy plots. Therefore, while non‐legacy and legacy plots showed a similar decrease of REW from June to July (by 13% and 12%, respectively), the decrease from July to August was larger in the legacy plots (by 16%) compared to the non‐legacy plots (by 9%). REW in the middle of the plots between spruce and beech showed a similar trend (Fig. 3b). In the non‐legacy plots, REW decreased from 44 ± 8% (June), to 23 ± 6% (July), to 14 ± 3% (August), which was significantly lower than in the legacy plots, decreasing from 75 ± 14% (June), to 55 ± 14% (July), to 38 ± 14% (August). Under beech, in contrast (Fig. 3c), there was no significant difference in REW between the non‐legacy and the legacy plots throughout the measurement period, although the non‐legacy plots had slightly lower REW in July and August (23 ± 4% and 13 ± 3%) compared to the legacy plots (33 ± 4 and 20 ± 3%).

**Fig. 4:**
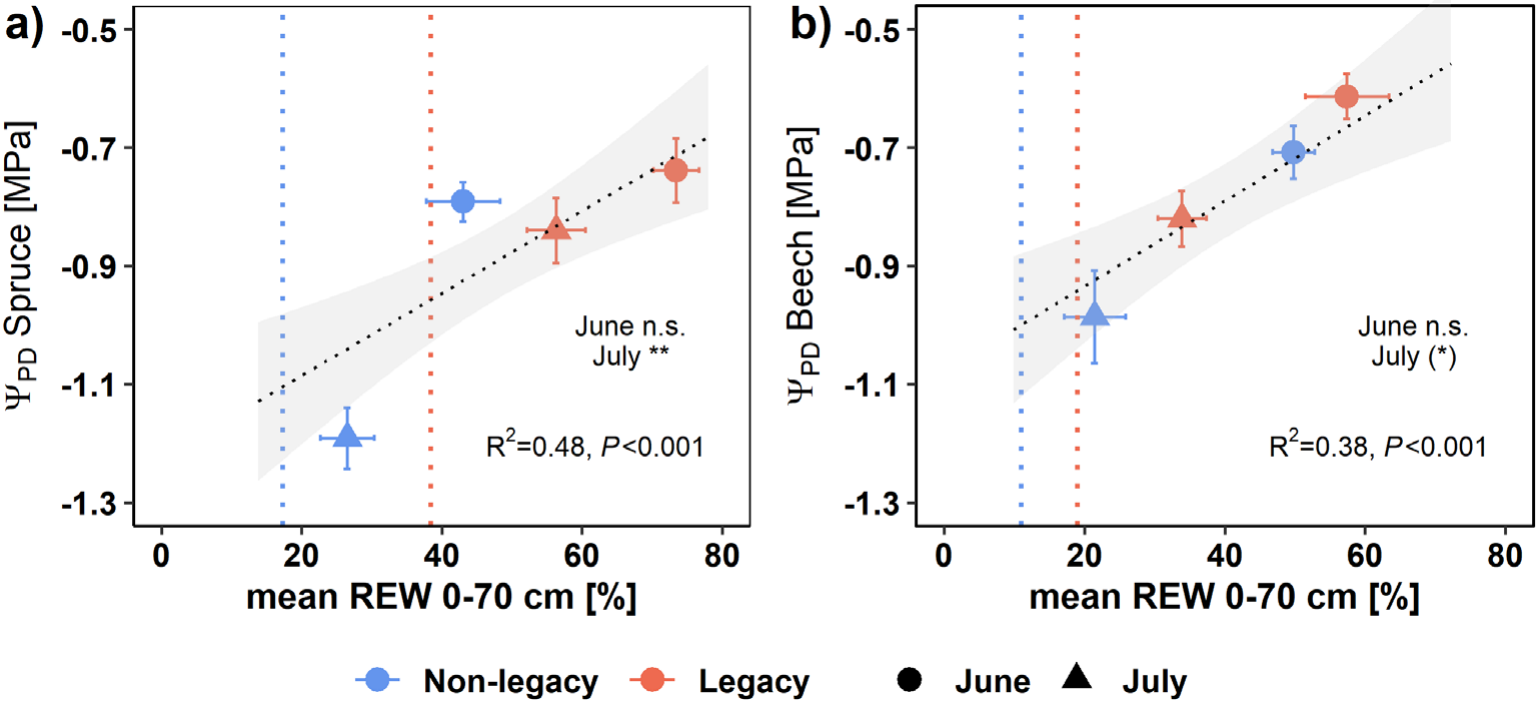
Predawn leaf water potential (Ψ_PD_) of spruce (a) and beech (b) against mean relative extractable water (REW) at 0–70 cm soil depths. For trees adjacent to TDR sensors positioned between spruce and beech (marked “x” in Fig. 1 as examples), average of REW Mix (Fig. 3b) and REW under the respective species (Fig. 3a or 3c) was used. For all other trees, REW under the corresponding species (Fig. 3a or 3c) was applied. Measurements of Ψ_PD_ in August could not be conducted due to sudden and unexpected rain during the night, followed by a long rain period, so no Ψ_PD_ data are available. Vertical dotted lines show mean REW during the August measurement campaign. Dotted slope is the linear regression (non‐legacy and legacy trees were fitted together) with grey area showing 95% confidence interval.

The Ψ_PD_ of non‐legacy spruce was −0.79 ± 0.03 MPa in June, which was similar to that of legacy trees at −0.74 ± 0.05 MPa (*P* > 0.8; Fig. 4a). In July, Ψ_PD_ of non‐legacy spruce significantly decreased to −1.19 ± 0.05 MPa (*P* < 0.001), which was significantly lower than that of legacy trees (−0.84 ± 0.05 MPa, *P* < 0.05). Comparably, non‐legacy beech trees significantly decreased Ψ_PD_ from June (−0.71 ± 0.04 MPa) to July (−0.99 ± 0.08 MPa, *P* < 0.01; Fig. 4b), while legacy beech trees showed a smaller and insignificant decrease (−0.61 ± 0.04 in June and − 0.82 ± 0.05 in July, *P* > 0.1). As a result, the difference between non‐legacy and legacy beech became larger from June to July (*P* < 0.1). For both species, there was a significant linear relationship between REW and Ψ_PD_ (Fig. 4; *P* < 0.001).

### Leaf gas exchange

**Fig. 5:**
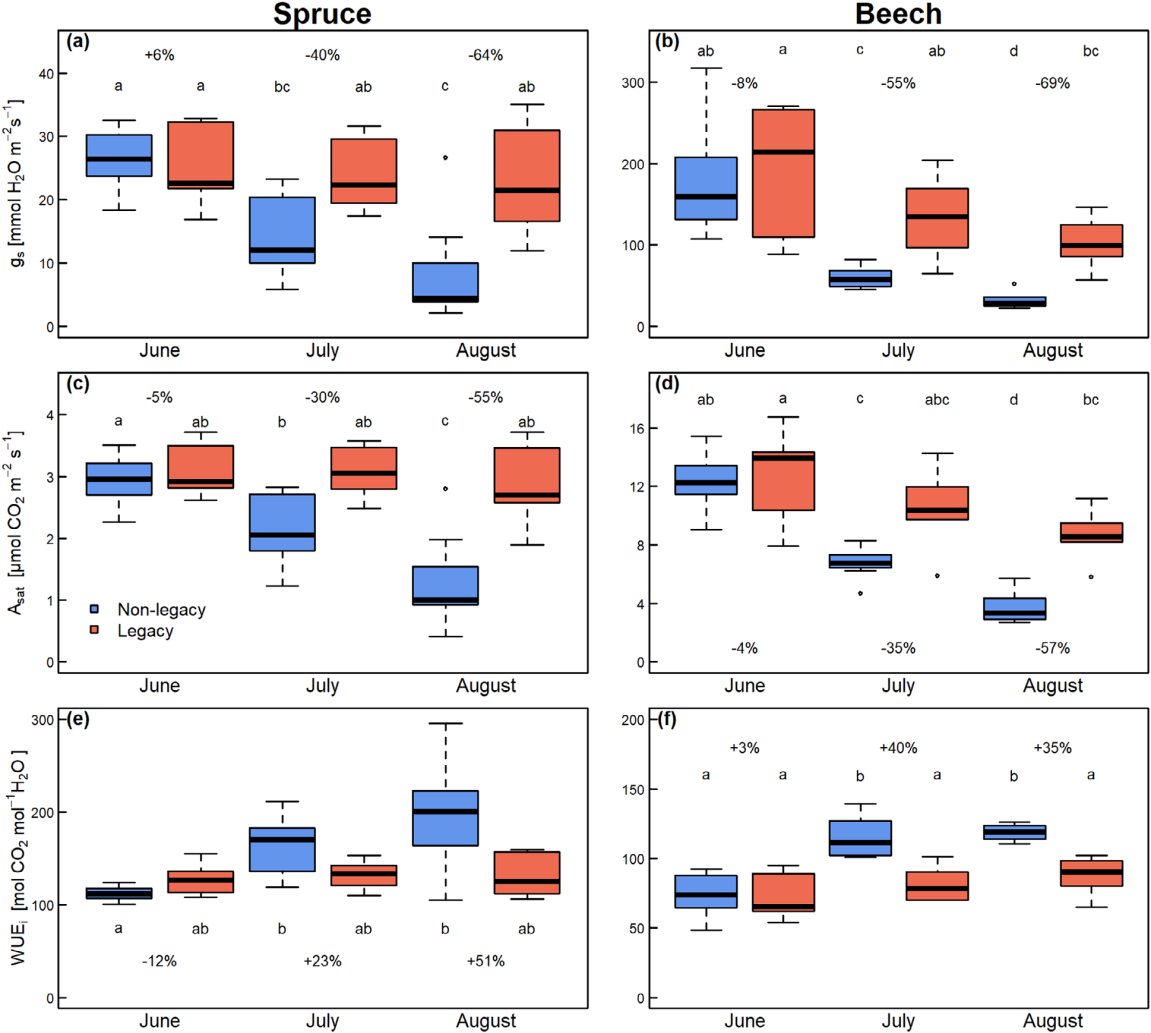
Stomatal conductance to water vapour (g_s_, a, b), light‐saturated CO_2_ assimilation rates (A_sat_, c, d), and intrinsic water use efficiency (WUE_i_, e, f) of non‐legacy (blue) and legacy (red) spruce and beech trees. Lowercase letters indicate significant differences among groups according to Tukey HSD. Values (%) indicate differences of mean of non‐legacy trees compared to mean of legacy trees. Thick lines in boxplots represent median.

Early in summer (June), non‐legacy spruce stomatal conductance (g_s_) was 26 ± 2 mmol m^−2^ s^−1^, similar to that of legacy spruce trees (Fig. 5a). In non‐legacy spruce, g_s_ significantly and gradually decreased in July and August to 14 ± 2 and 8 ± 3 mmol m^−2^ s^−1^, respectively. In contrast, g_s_ of legacy spruce remained constant throughout the year. Thus, non‐legacy spruce had 40% lower g_s_ in July than the legacy spruce, and 64% lower in August. Light‐saturated CO_2_ assimilation rates (A_sat_) showed a similar trend, with reduction in non‐legacy compared to legacy spruce, so that the difference between the treatments became gradually larger, reaching 30% and 55% in July and August (Fig. 5c). Intrinsic water‐use efficiency (WUE_i_) of non‐legacy spruce significantly increased from June to July and in August, reaching levels 23% higher in July and 51% higher in August than in legacy spruce, although these differences were not statistically significant (Fig. 5e). In contrast, in legacy spruce, WUE_i_ remained stable throughout the measurement period.

In beech, both non‐legacy and legacy trees significantly and gradually reduced their g_s_ from June to August, however, with a stronger decrease in non‐legacy compared to legacy trees (Fig. 5b). In June, g_s_ was similar between non‐legacy and legacy beech, at about 190 mmol m^−2^ s^−1^. Later during summer (July and August), non‐legacy beech showed a stronger decrease to 59 ± 5 and 31 ± 3 mmol m^−2^ s^−1^, respectively. This decrease was less pronounced in legacy beech, at 134 ± 21 in July to 102 ± 13 mmol m^−2^ s^−1^ in August. Likewise, the decrease in A_sat_ was less strong in legacy trees from June to August than in non‐legacy trees (Fig. 5d). Thus, similar to spruce, the difference between non‐legacy and legacy trees became larger from June to August, where the non‐legacy beech had 69% and 57% lower g_s_ and A_sat_ in August compared to the legacy beech. At the same time, intrinsic water‐use efficiency (WUE_i_) of non‐legacy beech significantly increased from June to July and in August, which was significantly higher than that of legacy beech in July (by 40%) and in August (by 35%). In contrast, in legacy beech, WUE_i_ remained constant throughout the measurement period (Fig. 5f).

### Sap flow density and daily water use of spruce

**Fig. 6:**
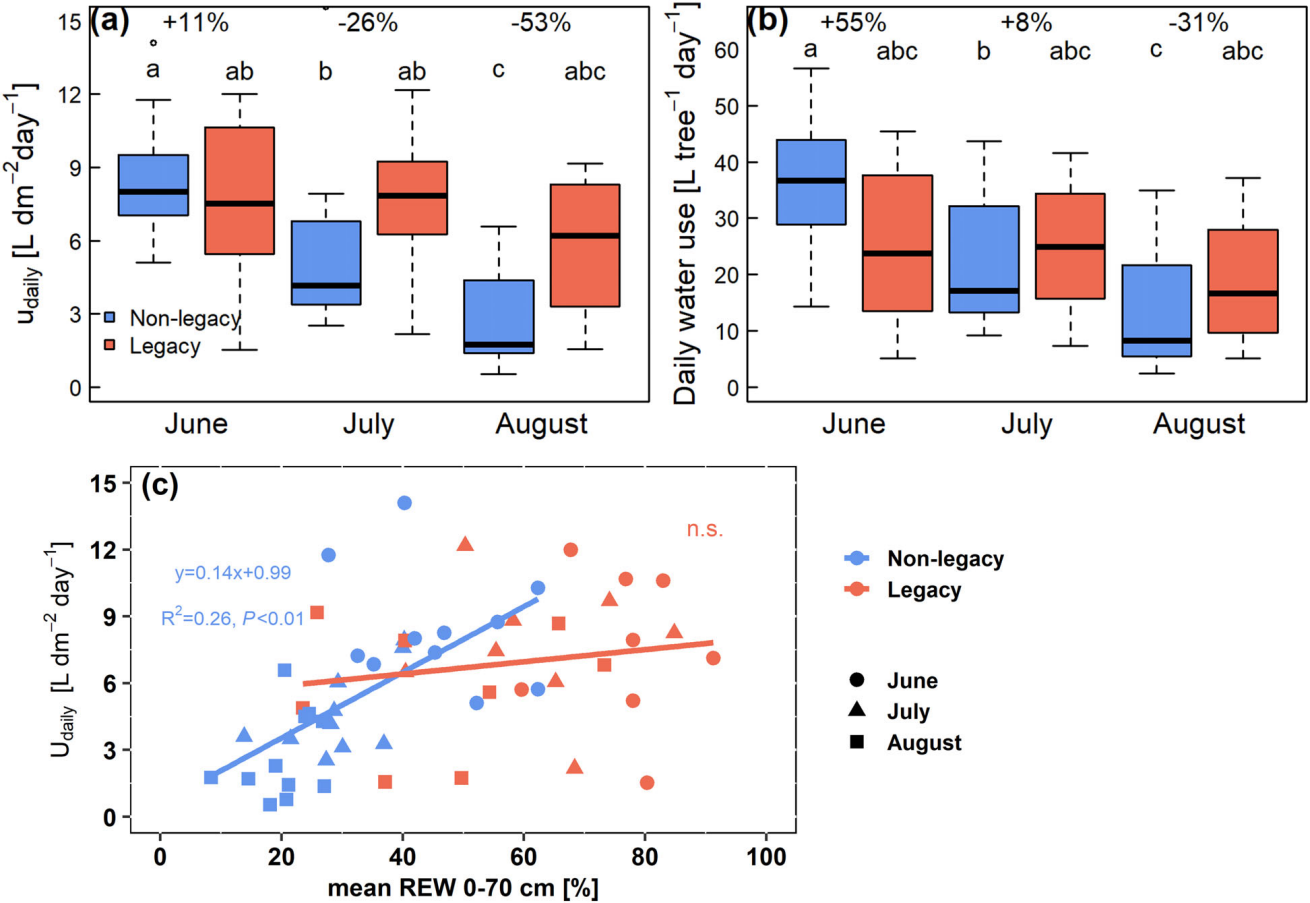
(a) Daily xylem sap flow density per day at outer 2 cm sapwood (u_daily_) and (b) whole‐tree daily water use in non‐legacy (blue) and legacy spruce (red) trees. (c) Scatter plot between u_daily_ and relative extractable water (REW) at 0–70 cm depth in non‐legacy (blue) and legacy (red) spruce trees. The u_daily_ was analysed on 7 sunny days without rainfall around the measurement campaign of leaf water potential and leaf gas exchange in each month. Lowercase letters indicate significant differences among groups according to Tukey HSD. Values (%) indicate differences of mean of non‐legacy trees compared to mean of legacy trees. Thick lines in boxplots represent median.

In June, mean sap flow density per day (u_daily_) was slightly higher in non‐legacy (8.5 ± 0.8 L dm^−2^ day^−1^) than in legacy (7.6 ± 1.2 L dm^−2^ day^−1^, Fig. 6a) spruce. Similar to photosynthesis parameters, u_daily_ of non‐legacy spruce trees significantly decreased over the growing season, to 5.6 ± 1.1 in July and 2.7 ± 0.6 L dm^−2^ day^−1^ in August, while u_daily_ of legacy spruce remained similar throughout the summer. This led to a lower u_daily_ of non‐legacy spruce of 26% and 53% compared to legacy spruce in July and August, respectively. Accordingly, the relationship between u_daily_ and REW was different between non‐legacy and legacy plots (Fig. 6c). Non‐legacy spruce showed a significant relationship between u_daily_ and REW, with a decrease in u_daily_ in parallel with decreasing REW. In contrast, legacy spruce showed no significant relationship between u_daily_ and REW. After including VPD as an additional independent variable, its influence on u_daily_ was stronger in the legacy spruce compared to the non‐legacy spruce, where u_daily_ of non‐legacy spruce was primarily dependent on REW (Fig. S3).

In June, daily water use of non‐legacy trees was 55% larger than that of legacy spruce (Fig. 6b). Throughout the summer, water use of non‐legacy spruce significantly decreased from 39 ± 5 to 27 ± 6 and 13 ± 3 L tree^−1^ day^−1^ in June, July and August, respectively. In contrast, legacy spruce did not significantly reduce their water use throughout the growing seasons (22 L tree^−1^ day^−1^ on average), leading to 31% lower water use in non‐legacy spruce by the end of the summer (August) compared to legacy spruce.

## DISCUSSION

The present study aimed to elucidate how the legacy in total leaf area following the previous long‐term drought (May 2014–June 2019) affects tree physiological responses to the next summer drought in 2022. The total leaf area of legacy spruce remained significantly reduced during the first three recovery years and was still 30% smaller than non‐legacy spruce 4 years after the long‐term drought. This slow recovery of spruce leaf area resulted in a higher REW during the natural summer drought of 2022, which led to a less physiological stress in legacy trees compared to non‐legacy trees.

### Total leaf area of legacy spruce was still reduced by 30% 4 years after long‐term drought

The 5 years of the long‐term drought experiment caused a significant reduction in needle length, shoot length, and thus total leaf area of spruce trees, which was accompanied, e.g. by a significant decrease in water use (Gebhardt *et al*. 2023; Hesse *et al*. 2024), and stem growth (Motte *et al*. 2023). At the end of the drought period in early summer 2019, legacy spruce trees only had one‐third of the total leaf area compared to non‐legacy trees. After the drought release in 2019, stomatal conductance and sap flow density recovered within 1 week and one growing season, respectively (Hesse *et al*. 2023). However, the needle and shoot length of legacy spruce remained significantly lower than those of non‐legacy trees for 2 years after the drought release (Fig. 2a,b). This is likely because shoot and leaf morphology of spruce not only depends on the climate conditions during the shoot/leaf development (Sutinen *et al*. 2015; Jansone *et al*. 2020; Zhu *et al*. 2022), but also is pre‐determined by the number of shoot/leaf units built during bud formation (Bollmark *et al*. 1995; Chen *et al*. 1996; Bréda *et al*. 2006). This may explain the decrease in shoot length of non‐legacy trees after the drier years of 2018/2019 (Schuldt *et al*. 2020) and the strong drought in 2022 at the study site (Fig. 3a). Because of the evergreen nature of spruce, i.e. retaining needles for 5–7 years at the study site (Figs. S1 and S2), the total leaf area of the legacy trees remained significantly reduced during the first recovery years and was still 30% smaller than that of non‐legacy trees 4 years after the drought release (Fig. 2c). Nevertheless, the difference in total leaf area between non‐legacy and legacy trees became smaller and was not significant in 2022 than at the end of the experimental drought treatment, because of the increase in number and area of current shoots in sun crowns after 2020 (Fig. 2d, Fig. S1), in addition to the recovery in shoot and needle length. Interestingly, the one legacy tree with the strongest leaf area reduction, i.e. 85%, did not show any leaf area recovery and, therefore, might have crossed a point‐of‐no‐return for leaf area loss, although this hypothesis should be tested with a larger number of replicates. The drought legacy on leaf area was paralleled by a delayed recovery of stem growth in spruce (Motte *et al*. 2023). Thus, reduced leaf area could be one reason for the delayed recovery of stem growth found in earlier studies (Anderegg *et al*. 2015; Peltier *et al*. 2016; Kannenberg *et al*. 2020; Li *et al*. 2023; Miller *et al*. 2023). In an irrigation experiment with pine trees growing on a naturally dry site, Zweifel *et al*. (2020) reported similar delayed responses in both crown morphology and stem growth after the end of the long‐term irrigation treatment. Accordingly, during the first year of recovery, 2019, legacy spruce preferentially allocated new photoassimilates belowground to rebuild fine roots (Hikino, Danzberger, Riedel, Hesse, *et al*. 2022), amplifying the delayed recovery of leaf and stem growth. Hence, 5 years of drought caused a long‐lasting drought legacy with reduced whole‐tree leaf area in spruce trees.

### Slow recovery of spruce total leaf area results in higher REW in soil and less physiological stress

At the beginning of the growing season, in April, REW was high (>75%) and similar in both legacy and non‐legacy plots (Fig. 3). Throughout the dry summer of 2022, however, REW was significantly higher in the legacy plots under spruce compared to the non‐legacy plots (Fig. 3), supporting the first hypothesis. This likely is a result of reduced water use of legacy spruce trees until the early summer related to slow recovery of the total leaf area (Fig. 2c). Moreover, reduced leaf area and resulting smaller crown size (Jacobs *et al*. 2021) may have increased stand precipitation by decreased interception, potentially contributing to the higher REW in the legacy compared to the non‐legacy plots. Because of the higher REW, leaf physiology and whole‐tree water use of the legacy spruce were less affected by the summer drought of 2022 compared to the non‐legacy spruce: Ψ_PD_, g_s_, A_sat_, and u_daily_ were all higher in the legacy compared to the non‐legacy spruce in July and August (Figs. 4, 5, 6), supporting the second hypothesis. Most impressive, legacy spruce almost maintained their whole‐tree water use throughout the summer drought of 2022, whereas that of non‐legacy spruce was significantly reduced by up to 70% (Fig. 6b). The calculation of whole‐tree water use is based on the sap flow density profile measured in the previous years (2019–2021), where the legacy spruce had lower sap flow density along the radial profile compared to the non‐legacy spruce (Gebhardt *et al*. 2023). Therefore, our calculation may underestimate water use of the legacy spruce if the sap flow density profile had (partially) recovered in 2022. Nevertheless, this clearly suggests that the whole‐tree water use of the legacy spruce was similar to or even higher but still had 30% smaller total leaf area in July and August compared to the non‐legacy trees, which is also supported by the larger decrease in REW from July to August in the legacy plots compared to the non‐legacy plots. This led to higher water availability per leaf area in the legacy spruce compared to the non‐legacy trees, as reflected in the higher g_s_ (Fig. 5a) as well as in the non‐significant relationship between u_daily_ and REW in the legacy spruce (Fig. 6c). This reduced physiological drought stress of legacy spruce may explain their increased stem growth in the drought year 2022 compared to the previous year 2021, whereas that of non‐legacy spruce decreased (Motte *et al*. 2023). Our results indicate that drought legacy can have a positive effect during the next drought on mature trees, as previously described for tree saplings and grass species (Nosalewicz *et al*. 2018; Pritzkow *et al*. 2021). Therefore, morphological drought responses may not only help trees to mitigate physiological stress during the current drought (Flexas *et al*. 2006; Ambrose *et al*. 2018; Bert *et al*. 2021; Lemaire *et al*. 2021), but can be also seen as acclimation for future drought events (Walter *et al*. 2013; Gessler *et al*. 2020).

In contrast to spruce, beech had a more anisohydric strategy, where g_s_ and u_daily_ were less affected during the 5 years of drought treatment (Hesse *et al*. 2024) and recovered faster after the drought release (Hesse *et al*. 2023). Furthermore, leaf area of beech trees was not significantly affected by the long‐term drought treatment (Hesse *et al*. 2024). Therefore, beech fully recovered whole‐tree water use already 1 year after the drought release (Gebhardt *et al*. 2023; Hesse *et al*. 2023), accompanied by faster recovery of stem growth in beech compared to spruce (Motte *et al*. 2023). Indeed, REW in beech was not significantly different between non‐legacy and legacy plots (Fig. 3c). Nevertheless, beech trees in the legacy plots, regardless of their distances from the spruce trees in the same plot, had higher Ψ_PD_, g_s_, and A_sat_, and a delayed drought effect compared to beech trees in the non‐legacy plots (Figs. 4 and 5), supporting the second hypothesis also for beech. Therefore, legacy in the spruce leaf area may also mitigated physiological stress of neighbouring trees. This result may indicate that beech trees expanded their roots (>5 m radius) towards spruce trees that had higher water availability, e.g. via active foraging over the four recovery years (Leuschner *et al*. 2001, 2004; Yanai *et al*. 2006; Nikolova *et al*. 2020; Zwetsloot & Bauerle 2021), since the total leaf area and thus likely the water use, had been smaller in legacy spruce compared to the non‐legacy trees during the four growing seasons after the drought release of 2019–2022 (Fig. 2c).

In conclusion, although slow leaf area recovery is associated with slow recovery of stem growth (Motte *et al*. 2023), this pronounced “negative” drought legacy effect on productivity can be advantageous for survival under future drought events. Our results may also help in understanding previous studies that used dendrochronology or remote sensing on other species and reported that stands experiencing a second drought showed less drought impact or mortality compared to stands that did not have a previous drought event (Bose *et al*. 2020; Norlen & Goulden 2023; Schmied *et al*. 2023). However, the summer drought of 2022 did not cause tree mortality in the study site, even in the non‐legacy plots, although strong physiological stress was evident. Therefore, it is still unclear whether the observed positive drought legacy might also delay tree mortality. Further studies should focus on examining to what extent the drought legacy in leaf area mitigates tree mortality under more severe and lethal droughts.

## AUTHOR CONTRIBUTIONS

KH and TEEG designed the study. KH, BDHe, TG, BDHa, MB and K‐HH contributed to data collection. KH analysed the data, with support from CB, BDHe, TG and BDHa. KH interpreted the data with support from BDHe, TG, BDHa and TEEG. KH wrote the manuscript and all authors revised the final manuscript.

## CONFLICT OF INTEREST

The authors declare that there is no conflict of interest.

## Supporting information


**Table S1.** Permanent wilting point (in vol.‐%) and soil saturation for plant available water (in vol.‐%) in each soil depth.
**Table S2.** VPD (vapour pressure deficit) and air temperature on the measurement days (7 days) of xylem sap flow during the daytime hours (8 am–8 pm, CET).
**Table S3.** Seasonal distribution of summed precipitation.
**Fig. S1.** Number of shoots of each needle age (N_s_, in *n* cm^−1^ needled branch length) in non‐legacy (blue) and legacy (red) spruce trees in sun crowns. N_s_ was counted on each tree twice after the growing season 2020 (left) and 2023 (right). To calculate the total leaf area in 2019 and 2020, N_s_ counted in 2020 was used. For the total leaf area in 2022, N_s_ counted in 2023 was used. The mean N_s_ from the two counting campaigns were used for the total leaf area in 2021.
**Fig. S2.** Number of shoots of each needle age (N_s_, in *n* cm^−1^ needled branch length) in non‐legacy (blue) and legacy (red) spruce trees in shade crowns. N_s_ was counted on each tree twice after the growing season 2020 (left) and 2023 (right). To calculate the total leaf area in 2019 and 2020, N_s_ counted in 2020 was used. For the total leaf area in 2022, N_s_ counted in 2023 was used. The mean N_s_ from the two counting campaigns were used for the total leaf area in 2021.
**Fig. S3.** (a) Three‐dimension scatter plot among u_daily_, mean relative extractable water (REW) at 0–70 cm depth, and vapour pressure deficit (VPD) in non‐legacy (blue) and legacy (red) trees. (b) Relative importance of REW and VPD for u_daily_ in non‐legacy and legacy spruce trees. The u_daily_ was analysed during 7 sunny days without rainfall around the measurement campaign of leaf water potential and leaf gas exchange in each month. Mean VPD and air temperature during the measurements are summarized in Table S2.
