# Supplementary material for: Drought legacy in mature spruce alleviates physiological stress during recurrent drought
Source: Plant Biol (Stuttg). 2025 May 16;28(3):637–48. doi: 10.1111/plb.70039 (PMC13089600; doi:10.1111/plb.70039)
Supplement: Supplementary file 1 — Table S1. Permanent wilting point (in vol.‐%) and soil saturation for plant available water (in vol.‐%) in each soil depth. Table S2. VPD (vapour pressure deficit) and air temperature on the measurement days (7 days) of xylem sap flow during the daytime hours (8 am–8 pm, CET). Table S3. Seasonal distribution of summed precipitation. Fig. S1. Number of shoots of each needle age (Ns, in n cm−1 needled branch length) in non‐legacy (blue) and legacy (red) spruce trees in sun crowns. Ns was counted on each tree twice after the growing season 2020 (left) and 2023 (right). To calculate the total leaf area in 2019 and 2020, Ns counted in 2020 was used. For the total leaf area in 2022, Ns counted in 2023 was used. The mean Ns from the two counting campaigns were used for the total leaf area in 2021. Fig. S2. Number of shoots of each needle age (Ns, in n cm−1 needled branch length) in non‐legacy (blue) and legacy (red) spruce trees in shade crowns. Ns was counted on each tree twice after the growing season 2020 (left) and 2023 (right). To calculate the total leaf area in 2019 and 2020, Ns counted in 2020 was used. For the total leaf area in 2022, Ns counted in 2023 was used. The mean Ns from the two counting campaigns were used for the total leaf area in 2021. Fig. S3. (a) Three‐dimension scatter plot among udaily, mean relative extractable water (REW) at 0–70 cm depth, and vapour pressure deficit (VPD) in non‐legacy (blue) and legacy (red) trees. (b) Relative importance of REW and VPD for udaily in non‐legacy and legacy spruce trees. The udaily was analysed during 7 sunny days without rainfall around the measurement campaign of leaf water potential and leaf gas exchange in each month. Mean VPD and air temperature during the measurements are summarized in Table S2. [file PLB-28-637-s001.docx]

**Supplementary materials**

**Drought legacy in mature spruce alleviates physiological stress during recurrent drought**

Kyohsuke Hikino, Benjamin D. Hesse, Timo Gebhardt, Benjamin. D. Hafner, Claudia Buchhart, Manuela Baumgarten, Karl-Heinz Häberle, and Thorsten E.E. Grams

Table S 1: Permanent wilting point (in vol.-%) and soil saturation for plant available water (in vol.-%) in each soil depth.

| Depths | Permanent wilting point [vol.-%]  (taken from Grams et al., (2021) | Soil saturation for plant available water [vol.-%]  (taken from Hesse et al., (2023) |
| --- | --- | --- |
| 0 - 7 cm | 7.4 ± 3.6 | 27.6 ± 5.0 |
| 10 – 30 cm | 13.5 ± 2.0 | 22.7 ± 3.8 |
| 30 – 50 cm | 19.1 ± 5.7 | 20.9 ± 3.6 |
| 50 – 70 cm | 25.8 ± 2.4 | 15.7 ± 3.5 |

Table S 2: VPD (vapour pressure deficit) and air temperature on the measurement days (7 days) of xylem sap flow during the daytime hours (8 am – 8 pm, CET).

| Month | VPD [kPa] | Air temperature [°C] |
| --- | --- | --- |
| June | 1.87 ± 0.03 | 25.5 ± 0.2 |
| July | 2.51 ± 0.04 | 26.7 ± 0.2 |
| August | 1.86 ± 0.03 | 24.7 ± 0.1 |

Table S 3: Seasonal distribution of summed precipitation.

| Precipitation (mm) | 2019 | 2020 | 2021 | **2022** |
| --- | --- | --- | --- | --- |
| Nov (previous year) - Feb | 212 | 197 | 157 | **184** |
| Mar - May | 169 | 96 | 204 | **128** |
| Jun - Aug | 227 | 349 | 387 | **159** |

**
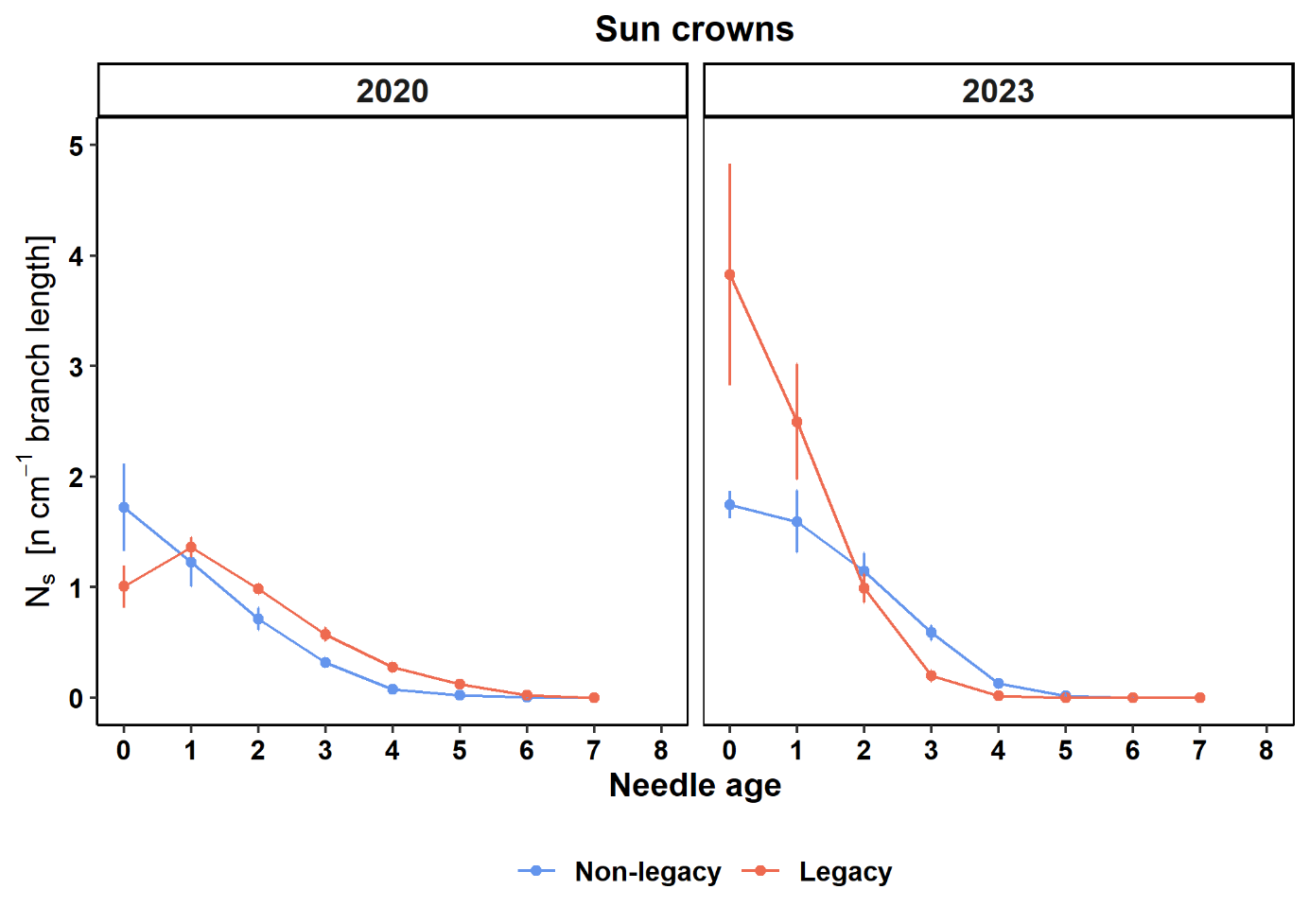
**

Fig. S 1: Number of shoots of each needle age (N_s_, in n cm^-1^ needled branch length) in non-legacy (blue) and legacy (red) spruce trees in sun crowns. N_s_ was counted on each tree twice after the growing season 2020 (left) and 2023 (right). To calculate the total leaf area in 2019 and 2020, N_s_ counted in 2020 was used. For the total leaf area in 2022, N_s_ counted in 2023 was used. The mean N_s_ from the two counting campaigns were used for the total leaf area in 2021.


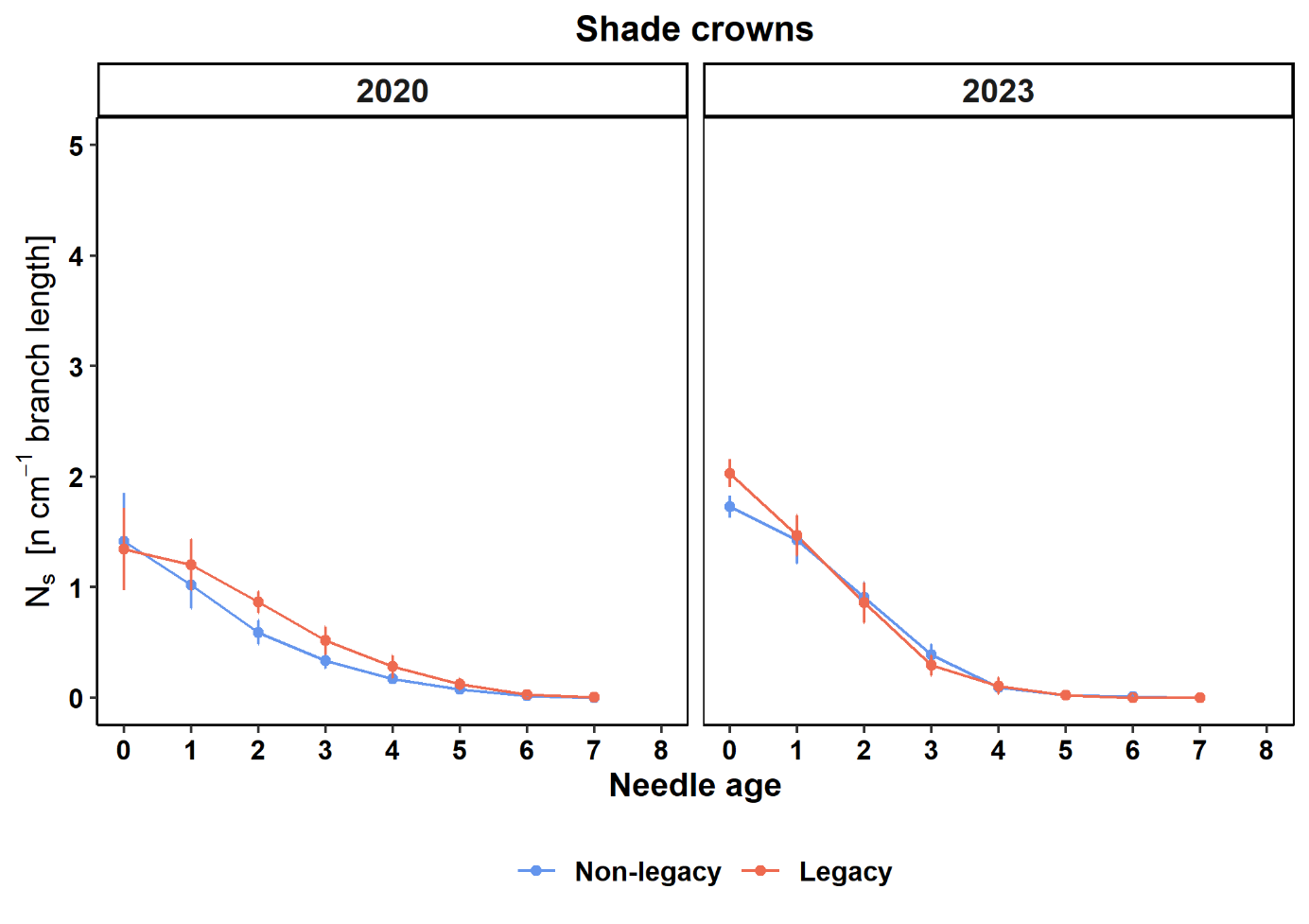


Fig. S 2: Number of shoots of each needle age (N_s_, in n cm^-1^ needled branch length) in non-legacy (blue) and legacy (red) spruce trees in shade crowns. N_s_ was counted on each tree twice after the growing season 2020 (left) and 2023 (right). To calculate the total leaf area in 2019 and 2020, N_s_ counted in 2020 was used. For the total leaf area in 2022, N_s_ counted in 2023 was used. The mean N_s_ from the two counting campaigns were used for the total leaf area in 2021.


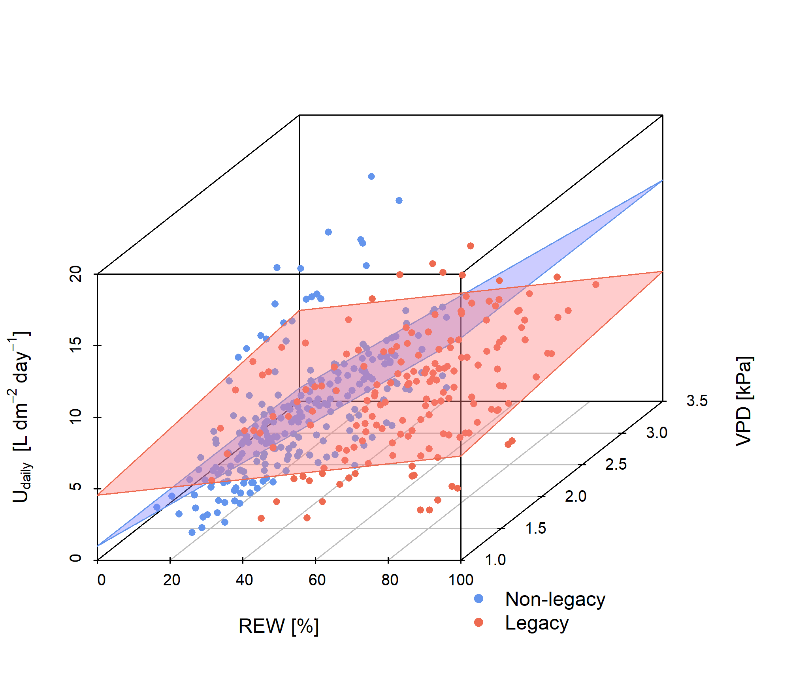

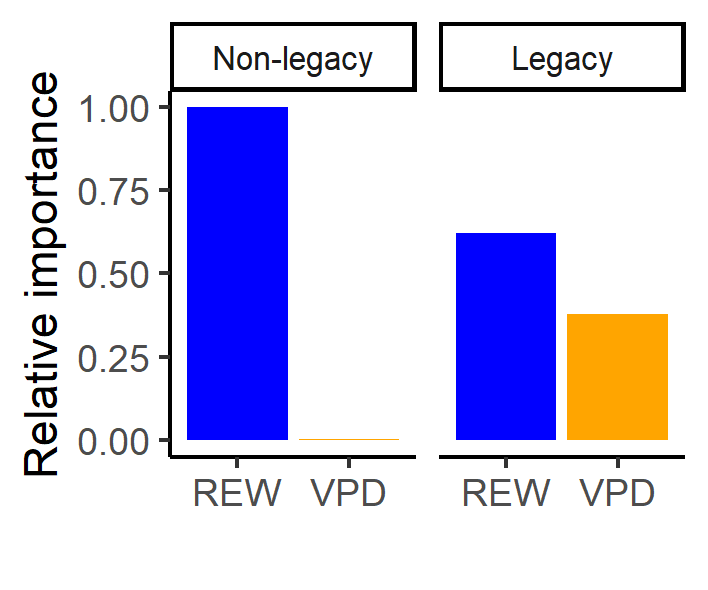


a)

b)

Fig. S 3: (a) Three-dimension scatter plot among u_daily_, mean relative extractable water (REW) at 0-70 cm depth, and vapour pressure deficit (VPD) in non-legacy (blue) and legacy (red) trees. (b) Relative importance of REW and VPD for u_daily_ in non-legacy and legacy spruce trees. The u_daily_ was analysed during 7 sunny days without rainfall around the measurement campaign of leaf water potential and leaf gas exchange in each month. Mean VPD and air temperature during the measurements are summarized in Table S2.

**References**

Grams, T. E. E., Hesse, B. D., Gebhardt, T., Weikl, F., Rötzer, T., Kovacs, B., Hikino, K., Hafner, B. D., Brunn, M., Bauerle, T., Häberle, K., Pretzsch, H., & Pritsch, K. (2021). The Kroof experiment: Realization and efficacy of a recurrent drought experiment plus recovery in a beech/spruce forest. *Ecosphere*, *12*(3), e03399. https://doi.org/10.1002/ecs2.3399

Hesse, B. D., Gebhardt, T., Hafner, B. D., Hikino, K., Reitsam, A., Gigl, M., Dawid, C., Häberle, K.-H., & Grams, T. E. E. (2023). Physiological recovery of tree water relations upon drought release—Response of mature beech and spruce after five years of recurrent summer drought. *Tree Physiology*, *43*(4), 522–538. https://doi.org/10.1093/treephys/tpac135
